# Self‐Healing and Shape‐Editable Wearable Supercapacitors Based on Highly Stretchable Hydrogel Electrolytes

**DOI:** 10.1002/advs.202201039

**Published:** 2022-06-26

**Authors:** Yizhou Zhao, Quanduo Liang, Samuel M. Mugo, Lijia An, Qiang Zhang, Yuyuan Lu

**Affiliations:** ^1^ State Key Laboratory of Polymer Physics and Chemistry Changchun Institute of Applied Chemistry Chinese Academy of Sciences Changchun 130022 P. R. China; ^2^ University of Science and Technology of China Hefei 230026 P. R. China; ^3^ State Key Laboratory of Electroanalytical Chemistry Changchun Institute of Applied Chemistry Chinese Academy of Sciences Changchun 130022 P. R. China; ^4^ Department of Physical Sciences MacEwan University Edmonton AB T5J4S2 Canada

**Keywords:** capacitance retention rate, hydrogel electrolytes, long‐term cycling durability, shape editability, wearable supercapacitors

## Abstract

Shape editability combined with a self‐healing capability and long‐term cycling durability are highly desirable properties for wearable supercapacitors. Most wearable supercapacitors have rigid architecture and lack the capacity for editability into desirable shapes. Through sandwiching hydrogel electrolytes between two electrodes, a suite of wearable supercapacitors that integrate desirable properties namely: repeated shape editability, excellent self‐healing capability, and long‐term cycling durability is demonstrated. A strategy is proposed to enhance the long‐term cycling durability by utilizing hydrogel electrolytes with unique cross‐linking structures. The dynamic crosslinking sites are formed by quadruple H bonds and hydrophobic association, stabilizing the supercapacitors from inorganic ion disruption during charge–discharge processes. The fabricated supercapacitors result in the capacitance retention rates of 99.6% and 95.8% after 5000 and 10 000 charge–discharge cycles, respectively, which are much higher than others reported in the literature. Furthermore, the supercapacitor sheets can be repeatedly processed into various shapes without any capacitance loss. The supercapacitors exhibit a 95% capacitance retention rate after five cutting/self‐healing cycles, indicative of their excellent self‐healing performance. To demonstrate real‐life applicability, the wearable supercapacitors are successfully used to power a light‐emitting diode and an electronic watch.

## Graphical abstract

A suite of wearable supercapacitors integrates the desirable properties of repeated shape editability, excellent self‐healing capability, and long‐term cycling durability. The supercapacitor sheets are repeatedly programmed into a series of shapes with no apparent loss in capacitance during the shape editing process. A strategy is proposed to enhance the long‐term cycling durability by utilizing hydrogel electrolytes with unique cross‐linking structures.

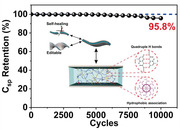

## 1 Introduction

Emerging wearable sensors and electronics have attracted considerable attention due to their applications in health monitoring, brain–computer interfaces, and robots.^[^ ^1^, ^2^, ^3^, ^4^, ^5^, ^6^ ^]^ These flexible devices are often used on moving bodies or subjects with arbitrary shapes. As such exploring novel energy powering devices that satisfy the requirements of wearable electronics is urgently needed. Supercapacitors are an important category of energy storage devices, which exhibit high power densities, fast charging and discharging, and fire safety.^[^ ^7^ ^]^ However, traditional supercapacitors have rigid architectures which limit their applicability to be embedded on flexible wearable devices. In recent years, most solid‐state supercapacitors have been fabricated using hydrogel electrolytes, which are flexible and exhibit excellent electrochemical performance.^[^ ^8^, ^9^, ^10^ ^]^ Due to their chemical stability and electrochemical inertness, polyvinyl alcohol (PVA)‐based electrolytes are the most commonly used polymeric substrates.^[^ ^11^ ^]^ To achieve high ionic conduction, a widely used strategy is to incorporate inorganic ions (e.g., acids, bases, metal ions, etc.) into the PVA‐based electrolytes.^[^ ^12^, ^13^, ^14^ ^]^ However, inorganic ions disrupt the H bonds between PVA chains and H_2_O, leading to intramolecular H bond formation that results in the dehydration and aggregation of PVA‐based electrolytes.^[^ ^15^ ^]^ The aggregation restricts the mobility of PVA segments, leading to poor self‐healing performance and limited stretchability. To overcome this issue and obtain self‐healing PVA‐based electrolytes, approaches such as dynamic cross‐linking based on H bonds, electrostatic interactions, and borate ester bonds have been used.^[^ ^16^, ^17^, ^18^, ^19^ ^]^ However, dynamic cross‐linking based on H bonds and electrostatic interactions are prone to disruption by inorganic ions during massive charge–discharge cycles, inadvertently changing the ion conduction path which reduces the supercapacitors capacitance retention. Relatively little research has been conducted to investigate hydrogel electrolytes with the ability to resist interruption by inorganic ions for supercapacitor applications.

To lend wearable supercapacitors to adapt to arbitrary configurations on the body surface, aside from high flexibility and self‐healing performance, shape editability is highly desirable. However, most of the flexible supercapacitors have static predesigned architectural configurations such as wave, bridge island, and fiber shapes.^[^ ^20^, ^21^, ^22^, ^23^, ^24^ ^]^ In addition, the predesigned strategy involves complex assembly processes and the unalterable postfabrication static structures limit the scope of wearable supercapacitors applications. Limited research has been conducted on the development of dynamic shape editable supercapacitors. The few studies include Chen and co‐workers who fabricated an editable supercapacitor by sandwiching a PVA gel electrolyte between two MnO_2_ nanowires/carbon nanotube (CNT) films.^[^ ^25^ ^]^ The MnO_2_ nanowire/carbon nanotube films were coated by nanocellulose fibers to enhance the mechanical properties of electrodes and to prevent supercapacitor from short‐circuiting during the folding, twisting, and stretching shape editing process. The supercapacitor sheet was fabricated into honeycomb‐like and pyramid pop‐up structures by linear pattern cutting. A similar strategy was utilized by Qu and co‐workers to achieve editable supercapacitors with arbitrary pre‐designed multidimensional configuration.^[^ ^26^ ^]^ 1D to 3D configurations could be prepared by laser cutting a supercapacitor sheet that was fabricated by sandwiching filter paper between reduced graphene oxide (rGO) films. Another strategy was reported by Sun and co‐workers who used poly(ethylene oxide) electrolyte and CNT film electrodes on a shape‐memory polyurethane (PU) substrate to fabricate a sheet‐like supercapacitor with versatile shape editability.^[^ ^27^ ^]^ Notwithstanding the few demonstrations, further research is needed in the nascent area of flexible, self‐healing, and shape‐editable wearable supercapacitors.

From the materials perspective, two factors play key roles in the performance of editable and self‐healing supercapacitors, that is, i) interface stress between hydrogel electrolytes and electrodes during shape editing, and ii) the characteristics of the crosslinking sites within hydrogels. The interface stress results from the interfacial physical‐chemical mismatch between flexible hydrogels and rigid electrodes, which often leads to the delamination of electrodes and supercapacitor short‐circuiting during shape editing.^[^ ^25^ ^]^ Most self‐healing hydrogel electrolytes are cross‐linked by dynamic electrostatic interactions and H bonds, rather than covalent cross‐linkage.^[^ ^28^, ^29^ ^]^ However, the electrostatic interactions and H bonds can partially dissociate under high ionic strength and electric field environments, causing changes in crosslinked structure, ion conduction path, and thereby reducing long‐term cycling durability.^[^ ^30^ ^]^

**Figure 1:**
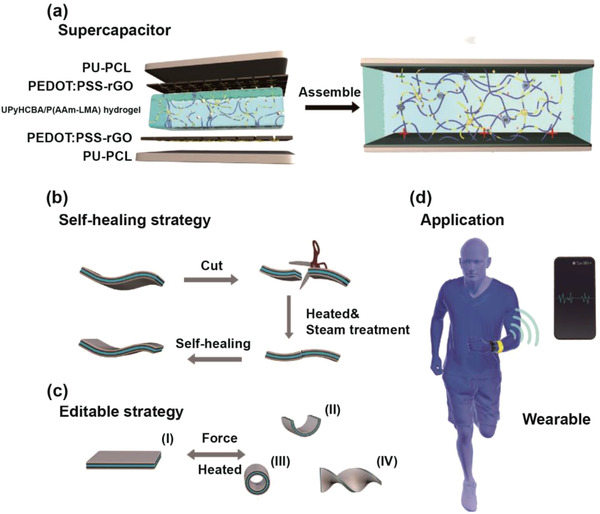
Schematic diagrams of a) the composition and structure of a supercapacitor, b) the self‐healing performance of supercapacitors, c) the shape‐editable performance of supercapacitors, and d) the application of wearable supercapacitors.

Herein, we demonstrate flexible supercapacitors with excellent self‐healing performance, repeatable shape editability, and high long‐term cycling durability (**Figure** **1**). The supercapacitors consisted of a highly stretchable self‐healing hydrogel electrolyte and electrodes on shape memory substrates. The hydrogels contain two kinds of dynamic crosslinking groups: 2‐ureido‐4‐pyrimidone (UPy) and lauryl groups. The UPy can form a self‐complementary dimer via quadruple H bonds with a dimerization constant of >10^6^ M^−1^.^[^ ^31^, ^32^ ^]^ The quadruple H bonds lead to exceptionally stable dimers, which can resist inorganic ions from dissociating the crosslinking sites in the hydrogel electrolytes. Through hydrophobic self‐assembly, the lauryl group can form hydrophobic crosslinking sites that enhance the mechanical and fatigue resistance of the hydrogel electrolytes, meanwhile tolerating inorganic ions damage. Both UPy and lauryl groups allow the hydrogel electrolyte excellent self‐healing performance while exhibiting super‐stretchability (strain > 12000%). These properties allow for electrolyte adaptability to electrode deformation, thus reducing the interface stress during shape editing. The flexible electrodes were prepared using poly(3,4‐ethylenedioxythiophene):poly(styrene sulfonate)/reduced graphene oxide composites (PEDOT:PSS‐rGO). The rGO‐based electrodes exhibit high specific surface area, long cycle life, and the electric double layer mechanism during the charge–discharge process. The typical low capacitance of pure rGO electrodes based capacitors attributed to reaccumulation of rGO layers can be prevented by the highly conductive PEDOT:PSS, which also enhances the electrodes electrochemical performance.^[^ ^33^ ^]^ Moreover, the PEDOT:PSS allows the PEDOT:PSS‐rGO electrode self‐healing performance by steam treatment. Finally, the use of PU derivative as a substrate affords self‐healing performance and repeatable shape editability. The assembled flexible supercapacitors demonstrated excellent self‐healing performance, repeatable shape editability, and high long‐term cycling durability. For example, a supercapacitor sheet was repeatedly programmed into a series of shapes such as “U,” “spiral,” and “ring” shapes, with no apparent loss in capacitance during the shape editing process. After five cutting/healing cycles, the supercapacitor exhibited a 95% capacitance retention compared to its original value. The supercapacitor demonstrated capacitance retention rates of 99.6% and 95.8% after 5000 and 10 000 charge–discharge cycles, respectively, which are much higher than those reported in the literature.^[^ ^19^, ^22^, ^28^, ^34^, ^35^, ^36^, ^37^, ^38^ ^]^ We further demonstrate the practical use of the fabricated supercapacitor in powering wearable electronic devices.

## 2 Results and Discussion

### 2.1 Preparation and Characterization of Hydrogel Electrolyte

**Figure 2:**
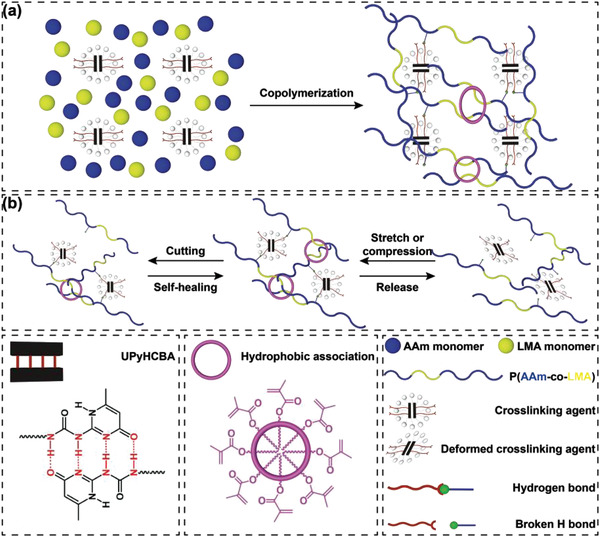
a) Schematic diagrams of hydrogel synthesis. b) Schematic mechanism of the self‐healing and fatigue resistance properties of the hydrogels.

**Figure 3:**
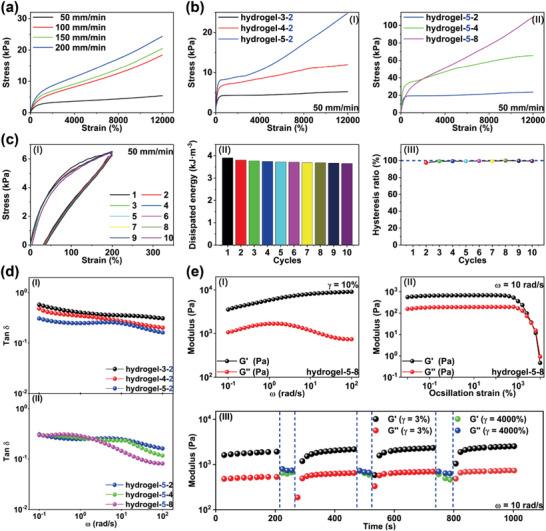
a) Stress–strain curves of hydrogel‐3‐2 at different stretching rates. b) Stress–strain curves of (I) hydrogel‐*x*‐2 and (II) hydrogel‐5‐y. c‐I) Hysteresis loop with tensile *λ* = 2 under cyclic loading with the recovery time of 1 min. II,III) Corresponding energy consumption and hysteresis ratios. d‐I) The tan*δ* of hydrogel‐*x*‐2 varies with the oscillation frequency. II) The tan*δ* of hydrogel‐5‐*y* with oscillation frequency. e‐I) Frequency dependence of *G*′ and *G*″ for hydrogel‐5‐8. II) Strain dependence of *G*′ and *G*″ for the hydrogel‐5‐8 at *ω* = 10 rad s^−1^. III) Repeated dynamic strain of hydrogel‐5‐8 in step tests (*γ* = 3% or 4000%).

Initially, the hydrogels were synthesized by the copolymerization of acrylamide (AAm), lauryl methacrylate (LMA), and 4‐(6‐(3‐(6‐(4‐methyl‐4‐oxa‐1,4‐dihydropyrimidin‐2‐yl) ureido) hexylcarbamoyloxy) butyl acrylate (UPyHCBA) (**Figure** **2**a). The UPy was attached to the hydrogel networks by a hydrophobic connector in UPyHCBA (Figure S1, Supporting Information). The hydrophobic connector can provide a hydrophobic microphase for the UPy dimers formed by quadruple H bonds, which further enhance the stability of the UPy dimers. The lauryl groups form dynamic crosslinking sites in the hydrogels by hydrophobic association. The hydrogels were denoted as hydrogel‐*x*‐*y*, where the *x* and *y* represent the mass fractions of the UPyHCBA and lauryl groups, respectively. The chemical structure of hydrogel‐3‐2 has been characterized by Fourier‐transform infrared spectrum (FTIR) that shows corresponding characteristic peaks, as shown in Figure S2 (Supporting Information). Excellent stretchability and fatigue resistance are essential to maintain the stable performance of supercapacitors during the shape editing processes and applications. The prepared hydrogels were cut into rectangular sheets for tensile testing. As shown in **Figure** **3**a, the hydrogels showed excellent stretchability. The tensile strain at fracture exceeded 12000%. The stress increased with the strain rate demonstrating a typical viscoelastic behavior. The stress–strain curves of the hydrogels with different contents of cross‐linking groups were recorded to investigate the effect of the cross‐linking sites on the mechanical properties (Figure 3b‐I). When the UPyHCBA content of the hydrogel‐*x*‐2 increases from 3 to 5 wt%, the stress increases from 5.3 to 24.8 kPa. The lauryl content also produces the same influence on the stress, as shown in Figure 3b‐II. The stress of the hydrogel‐5‐*y* increases from 23.7 to 109.7 kPa as the lauryl content increases from 2 to 8 wt%. Both the UPyHCBA and lauryl groups enhance the mechanical strength of the prepared hydrogels. In addition, cyclic tensile and compression tests were performed to study the cross‐linking dynamics. Successive ten tensile testing cycles with a recovery time of 1 min between consecutive cycles were conducted on hydrogel‐5‐8 (Figure 3c). Hysteresis loops were evident in the ten tensile cycles, which indicate the effective energy dissipation by the destruction and reconstruction of quadruple H bonds and hydrophobic association.

The hysteresis loops nearly overlap for the ten tensile testing cycles, which implies that the hydrogels retain the same interior network structure during the testing. Figure 3c‐II shows the energy dissipation of the hydrogel during the ten test cycles. The dissipation energy for the first, second, and tenth cycles are 3.9, 3.8, and 3.7 kJ m^−3^, respectively. Figure 3c‐III shows the change in hysteresis ratios of hydrogel‐5‐8 during the ten cycles. The hysteresis ratio was defined as the hysteresis normalization of each deformation cycle divided by that of the first cycle. The hysteresis ratios for the second and tenth cycles were 97.5% and 93.9%, respectively. The hysteresis ratios exceeded 93.5% for the ten tensile cycles, which indicates fast cross‐linking dynamics and significant fatigue resistance of the hydrogel‐5‐8. The toughness recovery for two continuous cycles reaches above 97.5% within 1 min rest, which can be ascribed to the fast cross‐linking dynamics of quadruple H bonds and hydrophobic association. Meanwhile, consecutive compression tests were conducted to investigate the compression fatigue resistance of the hydrogels (Figure S3, Supporting Information). The hydrogel‐5‐8 was compressed to 50% of its original thickness for ten consecutive compression testing cycles. As shown in Figure S3a (Supporting Information), the loading–unloading curves exhibit significant hysteresis and almost overlap. All of the hysteresis ratios for the ten compression cycles were above 85% (Figure S3c, Supporting Information). The toughness recovery for two continuous compression cycles reached above 96.6% within 1 min rest, which indicates that the hydrogel had excellent compression fatigue resistance. The hydrogels show excellent stretchability and fatigue resistance, which is much higher than other hydrogels reported in the literature (Figure S4, Supporting Information).^[^ ^19^, ^32^, ^39^, ^40^, ^41^, ^42^, ^43^, ^44^, ^45^, ^46^ ^]^

To characterize the self‐healing performance, a hydrogel‐5‐8 was vertically bisected (Figure S5, Supporting Information), and the two pieces were held together for a certain time without any external energy. The stress‐strain curves of the healed samples were then recorded under the same conditions as those for pristine sample testing. The stress‐strain curve was comparable to that of the original sample after 5 h (Figure S5, Supporting Information), which demonstrates the self‐healing ability against mechanical damage. The self‐healing ability and fatigue resistance of the hydrogels were attributed to the reversible destruction and reconstruction of quadruple H bonds and hydrophobic association (Figure 2b). A qualitative self‐healing test was performed on a piece of hydrogel‐5‐8 by bisecting and holding the two pieces together for 40 s (Figure S5, Supporting Information). This process formed a strong bond between the two pieces that withstood significant stretch without breaking.

Oscillatory rheology tests were conducted on the hydrogels to further investigate the fatigue resistance, viscoelastic behavior, and self‐healing performance at 25 °C. The loss factor (tan*δ*) is an important parameter to evaluate fatigue resistance. The tan*δ* is defined as the ratio of loss modulus (*G*″) to storage modulus (*G*′). The frequency dependence of tan*δ* in the rotation speed (*ω*) range of 0.1–100 rad s^−1^ at a strain (*γ*) of 10% is shown in Figure 3d. The values of tan*δ* decreased as the UPyHCBA content increased (Figure 3d‐I). The hydrogel‐5‐*y* was then used to explore the influence of the lauryl group content on tan*δ*. As shown in Figure 3d‐II, the hydrogels exhibit similar tan*δ* values in the low *ω* range (0.1–2.5 rad s^−1^). However, the influence of lauryl groups on tan*δ* is observed in the *ω* range of 3.2–100 rad s^−1^. The greater numbers of lauryl groups lead to lower tan*δ* values in this rotation speed range. This phenomenon can be attributed to the fact that more UPyHCBA and lauryl groups form more cross‐linking sites and networks. The highly cross‐linked networks result in small tan*δ* values and excellent fatigue resistance. Figure 3e‐I shows the frequency dependence of *G*′ and *G*″ at a *γ* of 10%. *G*′ was always larger than *G*″ in the *ω* range of 0.1–100 rad s^−1^, which is a typical characteristic of viscoelastic materials. Based on the results of the strain amplitude sweep test shown in Figure 3e‐II, the *G*′ and *G*″ values of the hydrogel‐5‐8 were constant as the *γ* increased from 0.1% to 400%, and the value of *G*′ was larger than that of *G*″. The results indicate that the hydrogel was in a gel state. Upon further increase of the applied strain, the *G*′ and *G*″ decrease dramatically and intersect at the *γ* of 4000%, which is indicative of the critical point between the gel and sol states. The value of *G*″ was larger than that of *G*′ at the *γ* of >4000%, indicating that the hydrogel‐5‐8 was in a sol‐state and the hydrogel network was largely disintegrated. This phenomenon can be attributed to the disintegration of cross‐linking networks at high strains.

Subsequently, repeated dynamic strain step tests (*γ* = 3% or 4000%) were performed to evaluate the self‐healing performance of the hydrogel‐5‐8. As shown in Figure 3e‐III, *G*′ is larger than *G*″ at the *γ* of 3%, and the *G*′ curve crosses the *G*″ curve at the *γ* of 4000%. The results indicate that the interior crosslinking networks disintegrate at the *γ* of 4000%. When the *γ* returns to 3%, *G*′ and *G*″ quickly revert to the original values, indicative of the reformation of interior crosslinking networks within the hydrogels. The cross‐linked networks of the hydrogel‐5‐8 could be restored within 60 s, as shown in Figure 3e‐III. The aforementioned results indicate that the hydrogel‐5‐8 has excellent stretchability, fatigue resistance, and self‐healing performance, and as such could have unique performance as an electrolyte in supercapacitors.

### 2.2 Preparation and Characterization of Shape Memory Substrates and Electrode

**Figure 4:**
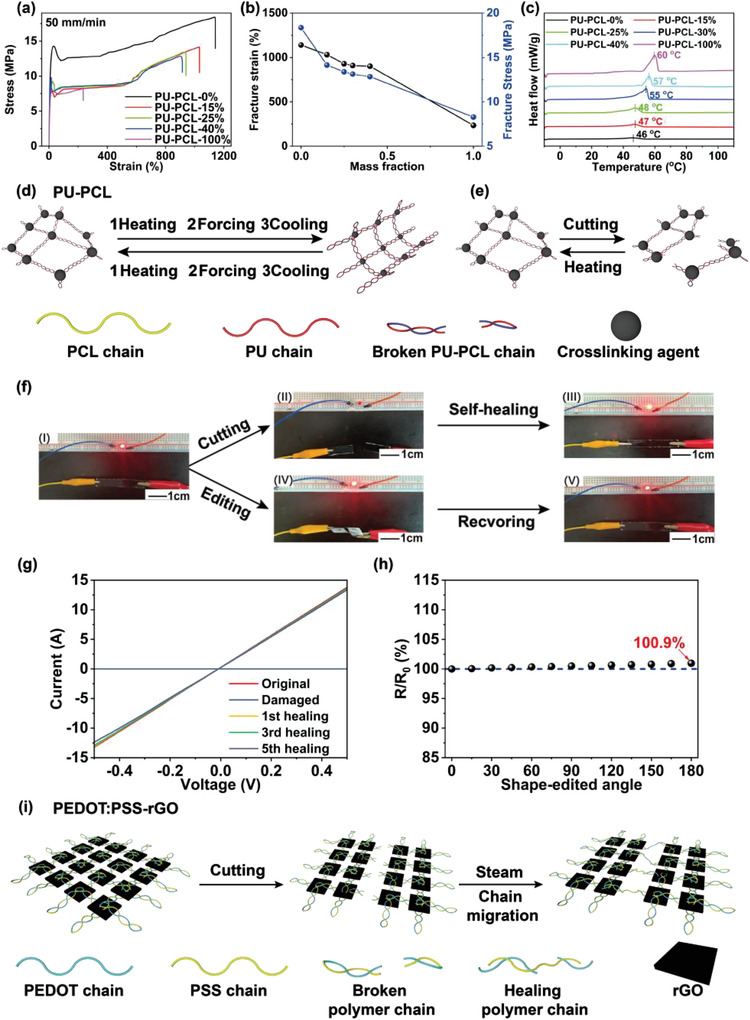
a) Stress–strain curves of PU, PCL, and PU‐PCL‐*x*%. b) Fracture stress and fracture strain variations of PU‐PCL‐*x*% with the PCL mass fraction. c) DSC curves of the PU, PCL, and PU‐PCL‐*x*%. For the DSC measurements, the heating curves of the second round were collected. Schematic mechanisms of d) shape‐editable and e) self‐healing process of PU‐PCL. f) Optical images of the PEDOT:PSS‐rGO electrode connected to a LED integrated circuit: (I) original, (II) after cutting, (III) after self‐healing, and (IV,V) after shape editing. g) The IV curves of the PEDOT:PSS‐rGO electrode after five cutting/self‐healing cycles. h) The normalized resistance of the PEDOT:PSS‐rGO electrode with a fixed angle from 0° to 180°. i) Schematic mechanism of the self‐healing process of PEDOT:PSS‐rGO electrode.

The substrate is an important component that provides shape editability and self‐healing performance for supercapacitors. In this work, polyurethane‐polycaprolactone composite (PU‐PCL) was used as substrate and the synthesis of PU has been shown in Figure S6 (Supporting Information). Initially, a linear PU was synthesized by the reaction of hexamethylene diisocyanate, 1,4‐butanediol, and polycaprolactone diol (*M*_n_ ≈ 4.5k). Then, the linear PU was crosslinked by triethanolamine to form PU networks (Figure S6, Supporting Information). A linear PCL (*M*_n_ ≈ 80k) was added to the PU solution under intense stirring to obtain a PU‐PCL composite. Sheet‐like PU‐PCL composite substrates were obtained by a casting method. The substrates were denoted as PU‐PCL‐*x*%, where the *x* represents the mass fractions of PCL. PU‐PCL‐15% was characterized by FTIR spectra to verify the functional components, as shown in Figure S7 (Supporting Information). The mechanical properties and melting transition temperature (*T*_m_) of the PU‐PCL composites could be controlled by adjusting the mass ratio of PU to PCL (**Figure** **4**). In comparison to PU, the PU‐PCL composite exhibits decreasing mechanical strength and flexibility due to the crystalline and brittle characteristics of PCL. All the PU‐PCL composite sheets exhibit the breaking stress of 12–13 MPa and the breaking strain of 800–1100% in the PCL content range of 15–40 wt%, which meet the mechanical requirements for supercapacitor substrates (Figure 4a,b). The melting transition of the substrates was characterized by differential scanning calorimetry (DSC) (Figure 4c). The PU and PCL exhibit a *T*_m_ of 46°C and 60 °C, respectively. The PU‐PCL composite substrates show a single *T*_m_ in the range of 47–57 °C. The *T*_m_ of pure PU or PCL does not appear in the DSC curves of the PU‐PCL‐*x*% substrates, indicative of the good molecular segment compatibility between the PU and PCL. The dynamic mechanical analysis (DMA) has been performed to investigate the thermomechanical property of PU‐PCL‐15%, as shown in Figure S8 (Supporting Information). It shows a storage modulus of 330 MPa at room temperature that starts to decrease at 45 °C and plateaus at 60 °C (7.0 MPa). This result indicates that the PU‐PCL composite can maintain satisfactory mechanical strength and structural integrity at 80 °C.

The configuration editability was investigated by programming the PU‐PCL sheets into various shapes, such as “U,” “ring,” and “spiral” shapes, at 65 °C (>*T*_m_). These temporary shapes were fixed by cooling to room temperature. The shape‐edited PU‐PCL sheets could be restored to their original shape when heated up to above 65 °C. The shape fixing rate (*R*_f_) and shape recovery rate (*R*_r_) are defined to evaluate the shape editability of PU‐PCL sheets.^[^ ^47^, ^48^ ^]^ The *R*_f_ shows the ability of a material to maintain a mechanical deformation during a shape editing process, which can be calculated by Equation S1 (Supporting Information). The *R*_r_ is used to quantify the ability of a material to memorize its permanent shape, which can be obtained using Equation S2 (Supporting Information). Cyclic thermomechanical experiments were employed by DMA to measure the *R*_f_ and *R*_r_ of the PU‐PCL composites (Figure S9, Supporting Information). The PU‐PCL sheets exhibit the *R*_f_ of 96.1%–91.7% and *R*_r_ of 99.3%–94.2% that are dependent on the PCL contents. The results indicate that the PCL component causes slightly inferior shape editability of the PU‐PCL sheets. The excellent editability can be attributed to the stable network and reversible switching segments. At the temperature of > *T*_m_, the PCL segments are mobile and the shapes of the PU‐PCL sheet became processable. When cooling to room temperature, the restricted movement of the PCL segment resulted in freezing the designed configuration. The stable cross‐linking network of PU enables PU‐PCL sheets to return to their permanent shape.

The self‐healing performance of the PU‐PCL substrates was investigated, as shown in Figure S10 (Supporting Information). A sheet was bisected using a blade, and the two pieces were then held together at 80 °C. The fractured PU‐PCL pieces were connected again, and the bending performance without any fracture was conducted in the damaged area. The healing efficiency is defined as the ratio of the tensile strength of a healed sample to that of its original counterpart. The healing efficiency of the PU‐PCL‐15% reached 73.6% after a 2 h treatment at 80 °C (Figure S10a, Supporting Information). These results indicate that the PU‐PCL composites could self‐heal after mechanical damage. To explore the effect of PCL content on self‐healing capability, the stress–strain tests were performed on PU‐PCL sheets after 2 h healing (Figure S10, Supporting Information). When the PCL content increases from 15 to 40 wt%, the healing efficiency increases from 73.6% to 91.8%. The results suggest that the PCL component conduces to the self‐healing performance of PU‐PCL composites. The shape editability and self‐healing performance can be attributed to the influence of *T*_m_ on the movement of molecular segments (Figure 4d,e). At room temperature, molecular segments were frozen. When the PU‐PCL is heated up to *T*_m_, the movement of the molecular segments is initiated, allowing the PU‐PCL shape to change under an external force. Then, the PU‐PCL specimen was cooled to room temperature causing the movement of the molecular segments to freeze, resulting in the generation of a temporary shape. Although a higher temperature would induce the movement of molecular segments, the stable networks derived from physical/chemical cross‐linking in the PU‐PCL remained integrated. Therefore, when the PU‐PCL was heated up to *T*_m_, its original shape was restored due to the interior tension generated by the cross‐linking networks. Similarly, the movement of molecular segments led to entanglements that enable the PU‐PCL self‐healing performance.

After the preparation and characterization of the PU‐PCL substrate, PEDOT:PSS‐rGO electrode was prepared on the surface of the substrate. rGO has been used as an electrode material as it exhibits high capacitance and long cycle life stemming from the high specific surface area and electric double‐layer mechanism during the charge‐discharge process.^[^ ^49^ ^]^ However, the capacitance of pure rGO is usually limited by the accumulation of rGO layers, hence requiring the addition of other electroactive polymers to achieve high capacitance.^[^ ^33^ ^]^ Compared to pure PEDOT:PSS or rGO electrode, it has been reported that PEDOT:PSS‐rGO electrode with an rGO content of 20% can exhibit the maximum energy and power density.^[^ ^33^ ^]^ However, if the rGO content exceeds 20%, the supercapacitor is reported to exhibit decreased capacitance due to low conductivity. In this work, the PEDOT:PSS‐rGO with a rGO content of 20% was used as the electrode material in the supercapacitor fabrication. The PEDOT:PSS‐rGO electrode was deposited on PU‐PCL substrates at 80 °C. The high temperature (above *T*_m_) facilitated chain entanglement between the PEDOT:PSS and the PU‐PCL, which enhanced the stability of the electrode and led to a uniform electrode film on the PU‐PCL substrate. The scanning electron microscopy (SEM) images of the PEDOT:PSS‐rGO electrode show a rough surface and a laminated structure across the film (Figure S11a,b, Supporting Information). The rGO enhances the surface roughness and creates the electrode with a laminated structure, in contrast to the PEDOT:PSS structure. The PEDOT:PSS‐rGO electrode shows the thickness of ≈6 µm and the loading mass of 2.4 mg cm^−2^ (Figure S12a, Supporting Information). The PEDOT:PSS‐rGO electrode exhibited a high conductivity (270 S cm^−1^) and excellent self‐healing performance. To qualitatively investigate the self‐healing performance, a PEDOT:PSS‐rGO sheet was used as a conductor to connect with a LED (Figure 4f‐I). When the PEDOT:PSS‐rGO sheet was bisected by a blade, the LED was immediately extinguished (Figure 4f‐II). The damaged area was steamed at 80 °C for 10 min and the LED was illuminated again (Figure 4f‐III). The effect of shape editability on conductivity was also evaluated, as shown in Figure 4f‐IV),V. A PEDOT:PSS‐rGO sheet was used as a connector between the power source and the LED. When the PEDOT:PSS‐rGO sheet was processed into a spiral shape, no significant changes in brightness were observed. To conduct a quantitative study on the self‐healing and shape‐editable ability of the electrode, we recorded the current–voltage (IV) curves during the self‐healing and shape‐editing process of the electrode (Figure 4g). The IV curves of the electrode show the changes in the electrical conductivity after the self‐healing process. Although the electrode exhibited a slight conductivity decrease (<5%), the healed electrode still maintained excellent conductivity after five cutting/healing cycles. The conductivity recovery efficiency (*R*_c_) was defined as the ratio of the conductivity of the healed specimen to that of the original counterpart, and the values were in the range of 95–98% during the five cutting/healing cycles. The change in resistance of the electrode was marginal even with the increase of the bending angles (Figure 4h). When an electrode was folded from 0° to 180°, it exhibited only a 1% resistance increment, which evidences the stability in conductivity performance during the shape‐editing process. The excellent self‐healing performance stems from the synergistic effect of the segment movement of PEDOT:PSS and PU‐PCL. At 80 °C, the self‐healing behavior of the PU‐PCL sheet led to close contact between the two separated PEDOT:PSS‐rGO pieces. Under steam treatment, the PSS chains quickly absorb water molecules, which leads to a volume increase filling the damaged area. Although the conductive PEDOT chains are hydrophobic and water‐insoluble, the expansion behavior of PSS transports the PEDOT chains forming conductive paths in the damaged area and thereby recovering the conductivity of the electrode (Figure S11c, Supporting Information and Figure 4i). The high electrical conductivity and excellent self‐healing characteristics of PEDOT:PSS‐rGO electrodes are key to superior electrochemical performance for wearable supercapacitors.

Furthermore, the stability of the electrodes on PU‐PCL substrates was investigated during the shape‐editing and self‐healing processes. Figure S12 (Supporting Information) shows the cross‐sectional SEM images of the electrodes after 10 “U” shape‐editing cycles and 5 cutting/healing cycles, respectively. The electrode and substrate still maintain integrated interfaces and no separation was found from the SEM images, which demonstrate the excellent mechanical stability during shape‐editing and cutting/healing processes.

### 2.3 Fabrication and Characterization of Supercapacitors

**Figure 5:**
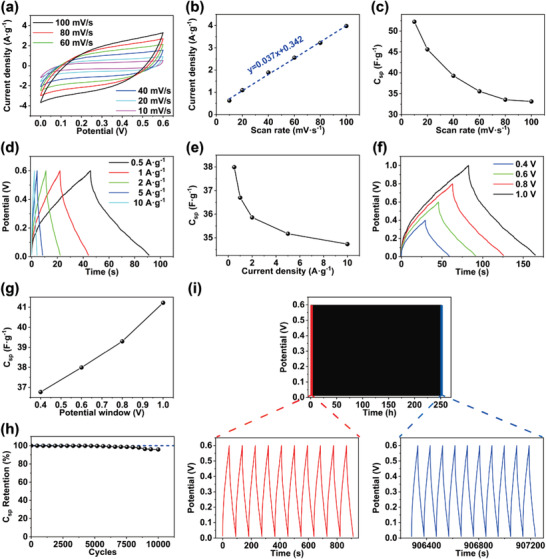
a) The CV curves of a supercapacitor at different scanning rates. b) The variation of the peak current of a supercapacitor with the scanning rate. c) The variation of the *C*_sp_ with scanning rates. d) The GCD curves of a supercapacitor at the current densities of 0.5–10 A g^−1^. e) The variation of *C*_sp_ per unit mass for different current densities. f) The GCD measurements for different potential windows at a current density of 0.5 A g^−1^. g) The variation of *C*_sp_ per unit mass for different potential windows. h) Capacitance retention of a supercapacitor after 10 000 charging and discharging cycles. i) GCD curves over 10 000 cycles.

All‐solid supercapacitors were fabricated by sandwiching the hydrogel‐5‐8 electrolyte between two PEDOT:PSS‐rGO electrodes on PU‐PCL‐15% substrates, which were encapsulated with Norland Optical Adhesive‐63 adhesive to prevent water evaporation. The hydrogel‐5‐8 electrolyte in the supercapacitor shows a thickness of ≈1 mm. The ionic conductivity of the hydrogel is 90 ms cm^−1^ determined by a four‐point probe apparatus. Cyclic voltammetry (CV) tests were conducted at a series of scanning rates to evaluate the supercapacitor electrochemical performance. As shown in **Figure** **5**a, all the CV curves were symmetric and had nearly rectangular shapes at the scanning rates of 10–100 mV s^−1^, suggesting a typical electric double‐layer capacitive (EDLC) behavior. The result demonstrates that the assembled capacitor could tolerate a fast charge–discharge process. An avalanche of ions diffuses from the hydrogel electrolyte into the electrode pores contributing to the pseudocapacitance effect. The diffusion of ions into the electrode holes can be inferred from the linear behavior (*R* ^2^ = 0.998) of current as a function of the scanning rate shown in Figure 5b. The specific capacitance (*C*_sp_) was calculated from the CV results using Equation S3 (Supporting Information). As shown in Figure 5c, when the scanning rate increased from 10 to 100 mV s^−1^, the *C*_sp_ decreased from 52.2 to 33.1 F g^−1^. This phenomenon results from the influence of ion diffusion at different scanning rates (Figure S13, Supporting Information). At a high scanning rate, ions migrate from the hydrogel electrolyte to the electrode surface with minimal diffusion into the inner space of the electrode. This process leads to fewer active sites participating in the electrochemical reaction, thereby reducing the insertion capacitance. The energy density also exhibits the same variation trend toward an increase in scanning rate. To investigate the charge transport process, electrochemical impedance spectroscopy (EIS) in the frequency range of 1 Hz to 10 kHz was conducted on the supercapacitor, as shown in Figure S14a (Supporting Information). The absence of a semicircle in the high‐frequency range indicates the high conductivity of the PEDOT:PSS‐rGO electrodes and the low charge transfer resistance during the charge‐discharge process. The straight line in the low‐frequency range corresponds to a slow diffusion process of ions into the electrodes indicative of good capacitive behavior (Figure S14b, Supporting Information). The low impedance value at 1 Hz (< 140 Ω) suggests that the ions could easily enter the PEDOT:PSS‐rGO electrode from the hydrogel electrolyte. The relaxation time constant of the supercapacitor was determined to be 0.04 s, which is the shortest time required to release all the energy, with an efficiency of >50% (Figure S14c, Supporting Information). Therefore, the supercapacitor shows great potentials for instantaneous delivery of high energy.

The galvanostatic charging and discharging (GCD) tests of the fabricated supercapacitor were conducted at various current densities (0.5–10 A g^−1^), as shown in Figure 5d. The GCD data exhibits triangular shapes, which confirm the EDLC behavior and show excellent reversibility during the charge–discharge process. The *C*_sp_, energy density, and power density were calculated from the GCD results using Equations S4–S6 (Supporting Information). The supercapacitor showed a *C*_sp_ of 38.0 F g^−1^ at the current density of 0.5 A g^−1^ and the *C*_sp_ value decreased as the current density increased (Figure 5e). The *C*_sp_ value increased slightly as the potential increased (Figure 5g). The supercapacitor exhibited an energy density of 1.9 Wh kg^−1^ with a power density of 150.0 W kg^−1^ at the current density of 0.5 A g^−1^. The variations of energy density and power density with the current density are shown in Figures S15 and S16 (Supporting Information). The increase in the current density leads to a decrease in the energy density and an increase in the power density. The charge–discharge rate of the supercapacitor shows an increase at higher current densities. This phenomenon results from the fact that the ions cannot enter deeply into the interior of the electrodes due to the shortened time. The coulombic efficiency of supercapacitors is defined as the ratio of the discharge capacity to the charge capacity. The coulombic efficiency of supercapacitors is 99.8%, which indicates the excellent energy release and negligible energy loss during the charging and discharging process. Long‐term cycling durability is one of the most important characteristics of supercapacitors in practical applications. Thus, 10 000 charge/discharge cycles were performed at a current density of 0.5 A g^−1^ to evaluate the cycling stability of the supercapacitors. As shown in Figure 5h,i, the supercapacitor exhibits capacitance retention rates of 99.6% and 95.8% after 5000 and10000 charge/discharge cycles, respectively, which are much higher than those reported in the literature (**Table** **1**). The excellent long‐term cycling durability can be attributed to the high stability of quadruple H bonds and hydrophobic association in the hydrogel electrolyte against inorganic ion disruption.

**Table 1:** The comparison of capacitance retention values with other supercapacitors

Electrodes	Electrolytes	Numbers	Retention	Refs.
Au@MnO_2_	LiClO_4_/PVA gel	500	85.5%	[34]
PEDOT: PSS‐rGO	Hydrogel‐5‐8	500	99.9%	This work
MoP@NC	PAM hydrogel	800	89.9%	[28]
PEDOT: PSS‐rGO	Hydrogel‐5‐8	800	99.9%	This work
PVA@PPy@AgNW	PVA hydrogel	3500	95.8%	[22]
PEDOT: PSS‐rGO	Hydrogel‐5‐8	3500	99.8%	This work
Ni‐Co‐N nanosheets	GO paper	5000	95.0%	[35]
Activated carbon/Zn	SPMA‐Zn hydrogel	5000	95.3%	[36]
PEDOT: PSS‐rGO	Hydrogel‐5‐8	5000	99.6%	This work
CNT films	Laponite/GO	10 000	93.0%	[19]
HPA‐rGO	PVA/H_2_SO_4_ gel	10 000	85.0%	[37]
Solvated graphene	PVA/H_2_SO_4_ gel	10 000	83.0%	[38]
PEDOT: PSS‐rGO	Hydrogel‐5‐8	10 000	95.8%	This work

### 2.4 Self‐Healing and Shape‐Editable Capability of Supercapacitors

**Figure 6:**
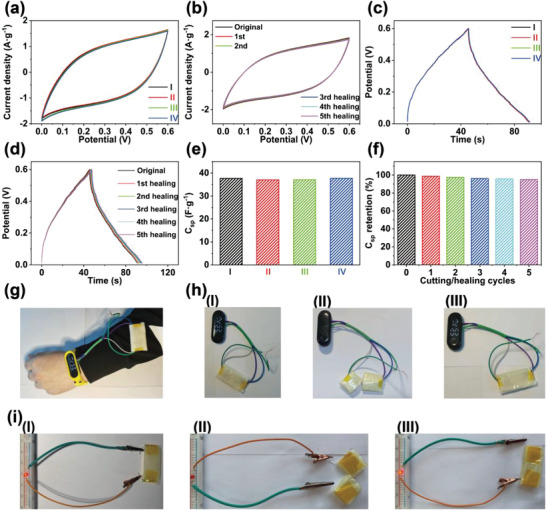
a) The CV curves of a supercapacitor in (I) original shape, (II) “U” shape, (III) “ring” shape, and (IV) “spiral” shape. b) The CV curves of a supercapacitor during five cutting/self‐healing processes. c) The GCD curves of a supercapacitor in (I) original shape, (II) “U” shape, (III) “ring” shape, and (IV) “spiral” shape. d) The GCD curves of a supercapacitor during five cutting/self‐healing processes. e) The *C*_sp_ values of a supercapacitor in (I) original shape, (II) “U” shape, (III) “ring” shape, and (IV) “spiral” shape. f) The *C*_sp_ retention of a supercapacitor during five cutting/self‐healing processes. g) Photograph of an electronic watch powered by a supercapacitor. h) Photographs of an electronic watch powered by a supercapacitor after (II) cutting and (III) healing. (i) Photographs of an LED powered by a supercapacitor after (II) cutting and (III) healing.

After the electrochemical characterization described above, the self‐healing and shape‐editable capability of the supercapacitors were tested. A supercapacitor sheet was processed into “U,” “ring,” and “spiral” shapes (Figure 1c‐II,IV). The CV and GCD curves of the shape‐edited supercapacitor overlapped with the original curves (**Figure** **6**a,c). No capacitance loss during the shape‐editing process was observed (Figure 6e). Self‐healing performance is very desirable for supercapacitors in practical applications. Herein, a supercapacitor was bisected, and the separated two pieces were butted together. The supercapacitor was held at 80 °C for 30 min while being steam treated. The supercapacitor self‐healed under this environment, and the healed supercapacitor could be bent and shape edited without breaking. Subsequently, CV and GCD tests were conducted on the healed supercapacitor to characterize the effects of the cutting/self‐healing process on the electrochemical performance, as shown in Figure 6b,d. After five cutting/healing cycles, the supercapacitor exhibited a 95.0% capacitance compared to its original value (Figure 6f).

To further investigate the stability of the supercapacitor during shape‐editing and self‐healing cycles, the supercapacitors were subjected to 20 “U” shape‐editing cycles and 10 cutting/self‐healing cycles, respectively. The supercapacitors exhibit the capacitance retention of 97.5% and 92.2% (Figure S17, Supporting Information), respectively, indicating excellent stability during the shape‐editing and self‐healing experiments. Electrochemical impedance spectroscopy (EIS) tests were conducted to investigate the impedance changes during the shape‐editing and self‐healing processes. The Nyquist plot and AC impedance plot of the supercapacitor after 20 “U” shape‐editing and 10 cutting‐healing cycles almost overlapped with that in the original state (Figure S18, Supporting Information). The supercapacitor exhibits only 3% and 8% increases in internal resistance after these shape‐editing and cutting‐healing processes, respectively. In order to further investigate the cycle stability of the supercapacitor after the shape‐editing and self‐healing processes, 10 000 charging–discharging cycles were performed on supercapacitors that underwent 20 “U” shape‐editing and 10 cutting/healing cycles, respectively (Figure S19, Supporting Information). The supercapacitors exhibit retention rates of 93.5% and 91.3% after the shape‐editing and cutting/healing cycles. The results indicate that the supercapacitors can maintain excellent electrochemical stability during the shape‐editing and cutting/healing tests. To demonstrate practical applicability, the supercapacitors were used to power an LED and a wearable electronic watch, as shown in Figure 6, Movies S1 and S2 (Supporting Information). The supercapacitor can drive the wearable electronic watch for 15 s. A charged supercapacitor sheet was twisted into “U” shape, “ring” shape, and “spiral” shape, yet it retained the ability to power an LED without any performance loss during the process (Figure 6 and Figure S20, Supporting Information). We first bend a supercapacitor 10 times. Then the supercapacitor shows the capability of lighting up an LED after the 10 bending cycles. Then the LED was powered by the supercapacitor when the supercapacitor was subjected to another five bending cycles. LED light does not show obvious changes in brightness during the bending process. This verifies that the supercapacitor retains a good function during the bending process.

The supercapacitor was bisected and then self‐healed to evaluate its energy output after the self‐healing process. When the supercapacitor was bisected, the LED light turned off, and the electronic watch did not display the time on the screen. The separated supercapacitor further exhibited the ability to power a LED and electronic watch after self‐healing. The aforementioned results prove the excellent shape editability and self‐healing performance of the supercapacitor during practical applications.

## 3 Conclusions

In summary, flexible supercapacitors with repeatable configuration editability and excellent self‐healing performance were prepared by sandwiching a hydrogel electrode between two PEDOT:PSS‐rGO electrodes on shape memory PU‐PCL substrates. This work demonstrates long‐term cycling durability of supercapacitors can be enhanced by utilizing special crosslinking sites in hydrogel electrolytes which can prevent inorganic ions interruption during charge–discharge processes. The increase in the long‐term cycling durability of the fabricated supercapacitors can be attributed to the quadruple H bonds formed by UPy dimers and hydrophobic association used as crosslinking sites in the hydrogel electrolyte. The wearable supercapacitors exhibited capacitance retention rates of 99.6% and 95.8% after 5000 and 10 000 charge–discharge cycles, respectively, superior to those reported in the literature. The dual dynamic crosslinking sites further endowed the hydrogel electrolytes with fast self‐healing capability, super‐stretchability, and excellent fatigue resistance. The shape memory PU‐PCL substrates provided the repeatable shape editability and self‐healing performance for the supercapacitors, which were attributed to the reversible changes in the movement of molecular segments caused by the glass–rubber transition behavior. The PEDOT:PSS‐rGO electrode also exhibited an excellent self‐healing capability and flexibility under steam treatment.

The fabricated supercapacitor could be repeatedly processed into desired shapes, such as “U,” “ring,” and “spiral” shapes, with no loss in specific capacitance. The excellent shape editability allows the supercapacitor to adapt to arbitrary configurations on the body or various surfaces, with excellent self‐healing abilities against external mechanical damage. When the supercapacitor was bisected, the two specimens could be self‐healed into an intact supercapacitor, retaining 97.8% capacitance. Even after five cutting/healing cycles, the self‐healed supercapacitor retained ≈95% capacitance. The supercapacitor with shape editability and self‐healing capability was successfully demonstrated in powering a LED and a wearable electronic watch, evidence of promise for practical energy storage applications.

## Conflict of Interest

The authors declare no conflict of interest.

## Supporting information

Supporting Information

Supplemental Movie 1

Supplemental Movie 2

## Data Availability

The data that support the findings of this study are available from the corresponding author upon reasonable request.
