# Supplementary material for: Self‐Healing and Shape‐Editable Wearable Supercapacitors Based on Highly Stretchable Hydrogel Electrolytes
Source: Adv Sci (Weinh). 2022 Jun 26;9(24):2201039. doi: 10.1002/advs.202201039 (PMC9405484; doi:10.1002/advs.202201039)
Supplement: Supplementary file 1 — Supporting Information [file ADVS-9-2201039-s003.pdf]

Supporting Information

Self-Healing and Shape-Editable Supercapacitors Based on Highly Stretchable Hydrogel Electrolytes

Yizhou Zhao, Quanduo Liang, Samuel M. Mugo, Lijia An, Qiang Zhang, Yuyuan Lu**

1. Materials

The following materials were obtained from commercial sources: polycaprolactone diol (M_w ~ 4.5k, Shenzhen Guanghua Weiye Co., Ltd.), polycaprolactone (PCL) (M_w ~ 80k, Procitan), poly(3,4-ethylenedioxythiophene)-poly(styrene sulfonate) (PEDOT:PSS) (1.5 wt%, Macklin), hexamethylene diisocyanate (HDI) (99%, Macklin), triethanolamine (TEA) (98%, Macklin), acrylamide (AAm) (99%, Macklin) , 2-amino-4-hydroxy-6-methylpyrimidine (MIC) (98%, Macklin), triethylamine (TEA, Macklin), tetramethylethylenediamine (TEMED) (99.5%, Aladdin), 4-hydroxybutyl acrylate (4-HBA) (95%, Aladdin), 1,4-butanediol (1,4-BDO) (99%, Tichai (Shanghai) Chemical Industry Development Co., Ltd.), dibutyltin dilaurate (DBTDL) (95%, Tichai (Shanghai) Chemical Industry Development Co., Ltd.), sodium dodecyl sulfate (SDS) (analytical grade, Beijing Chemical Plant), ammonium persulfate (APS) (analytical grade, Beijing Chemical Plant), sodium chloride (NaCl) (analytical grade, Xilong Science Co., Ltd.), petroleum ether (analytical grade, Xilong Science Co., Ltd.), chloroform ($CHCl_3$) (analytical grade, Xilong Science Co., Ltd.), and lauryl methacrylate (LMA) (97%, thermo

Fisher Scientific (China) Co., Ltd.). Norland Optical Adhesive 63 (NOA-63) (Norland Products Inc.)

2. Synthesis of 4-(6-(3-(6-methyl-4-oxo-1,4-dihydropyrimidin-2-yl)ureido)hexylcarbamoyloxy)butyl acrylate (UPyHCBA)

MIC (5.82 g, 50 mmol) and HDI (54.4 g, 0.32 mol) were refluxed at 100 ^{o}C for 20 h. The mixture was then added to 300 mL petroleum ether to obtain a white precipitate. The white precipitate was filtered with a Büchner funnel, washed with petroleum ether, and dried in a vacuum oven at 50 ^{o}C for 5 h to obtain 2-ureido-4-pyrimidone (UpyHI). Next, 0.40 mL TEA was added to a mixture of UPyHI (14.67 g, 50 mmol) and 4-HBA (7.92 g, 55 mmol) in 500 ml $CHCl_3$, and the mixture was allowed to react at 25 ^{o}C for 24 h. After the solvent was removed under reduced pressure, the residue was washed with excess acetone and dried to obtain a white solid product.

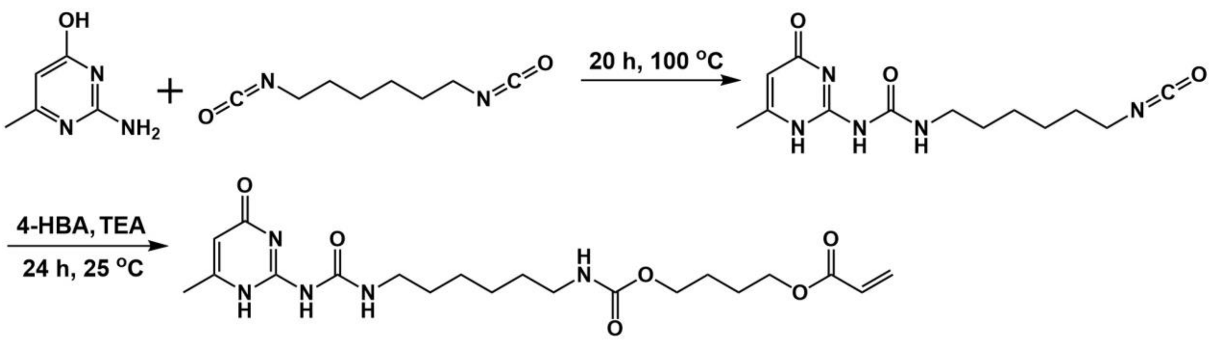

Figure S1. The synthesis of UPyHCBA.

3. Preparation of hydrogels

NaCl (1.169 g) and SDS (3.200 g) were dissolved in 40 ml distilled water to obtain a transparent solution. UPyHCBA (0.160 g) was added to the mixed solution, and the mixture was stirred on a hot plate at 35 °C for 4 h. The solution was then filtered with a 0.45 μm syringe filter to remove any remaining UPyHCBA particles in the solution. The solution was transferred to a beaker, and acrylamide (4.548 g) was added. Next, 150 μL TEMED and 200 μL APS were added to initiate the polymerization reaction. The polymerization reaction was allowed to proceed at 35 °C for 18 h to obtain a hydrogel sample.

4. Characterization of hydrogels

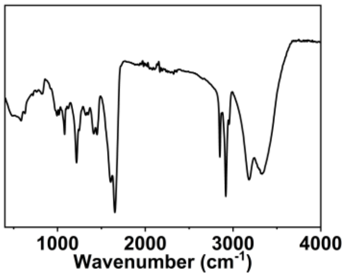

Figure S2. FTIR spectra of the hydrogel-3-2. The following characteristic peaks confirm the chemical structures: 3325 cm^{-1} (ν(-NH_2)), 3176 cm^{-1} (ν(OC-N-H)), 2916 and 2848 cm^{-1} (ν(CH_3, CH_2)), 1650 cm^{-1} (ν(CO-N)), 1606 cm^{-1} (ν(OC-N-H)), 1450 cm^{-1} (δ(CH_2)), 1218 cm^{-1} (ν(OC-O)), 1078 cm^{-1} and 987 cm^{-1} (ν(SO_4)).

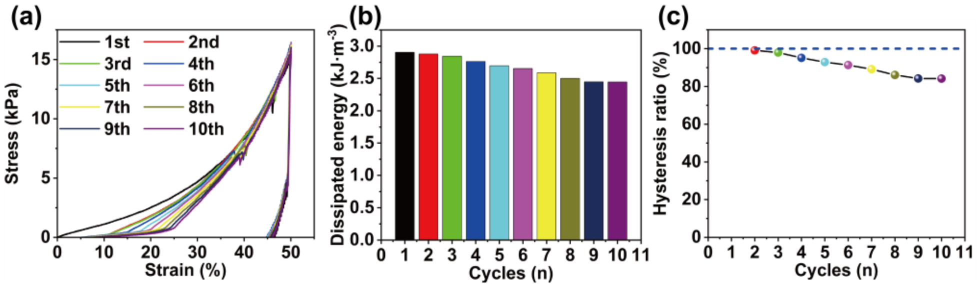

Figure S3. (a) Hysteresis loop of hydrogel-5-8 with compression λ = 0.5 during ten compression testing cycles. The recovery time between each cycle was 1 min. (b, c) The energy consumption and hysteresis ratio of hydrogel-5-8 during ten compression testing cycles.

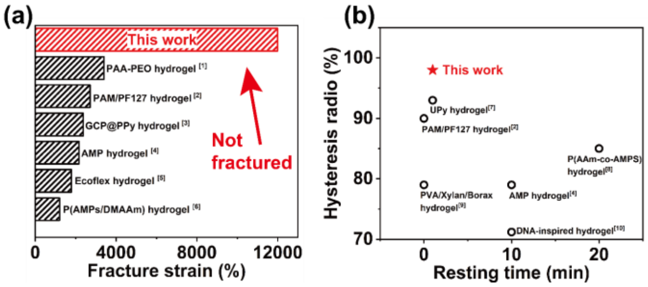

Figure S4. Comparison of stretchability and anti-fatigue properties of hydrogel-5-8 with those of other hydrogels. [1–10]

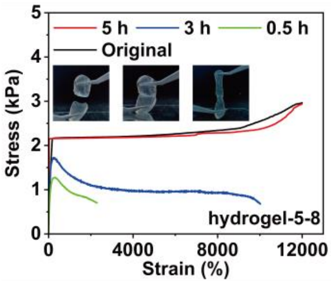

Figure S5. The stress–strain curves of hydrogel-5-8 during a self-healing process. The insets show the optical photographs of the self-healing process including fracture, contact, and self-healing.

5. Preparation of polyurethane–polycaprolactone (PU-PCL) substrate

The polycaprolactone diol (5 g, 1.25 mmol) was heated under vacuum at 120 ^{o}C to remove water until no bubbles were observed. The temperature was cooled to 60 ^{o}C, and HDI (0.5628 g, 3.35 mmol), DBTDL (10 mg), and THF (40 mL) were added. The reaction lasted for 24 h under an N_2 atmosphere. BDO (0.18 g, 2 mmol) was then added, and the reaction continued for another 24 h. Next, TEA (0.015 g, 0.1 mmol) was added to the system, and the reaction

continued for 12 h to obtain a PU-PCL solution. Under vigorous stirring, the PU-PCL solution was poured into a glass mold, and the solvent was evaporated at room temperature for 12 h to obtain a PU-PCL sheet. The PU-PCL sheets were further dried under vacuum at 60 °C and used as supercapacitor substrates.

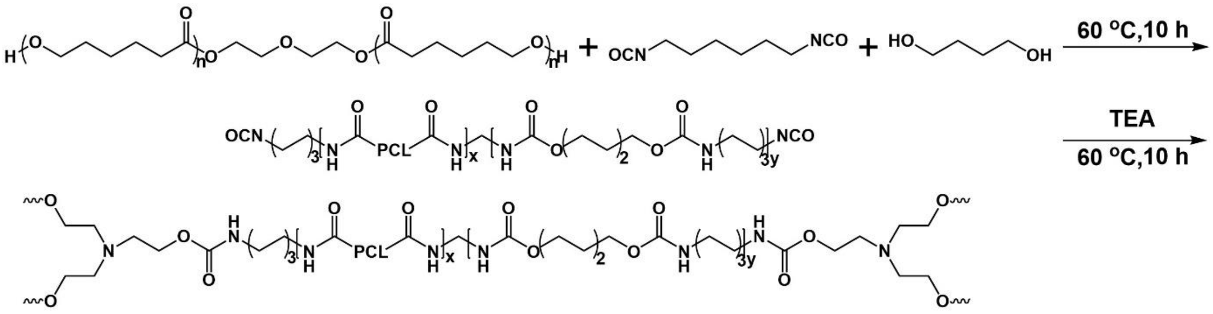

Figure S6. The synthesis of PU.

6. Characterization of PU-PCL substrate

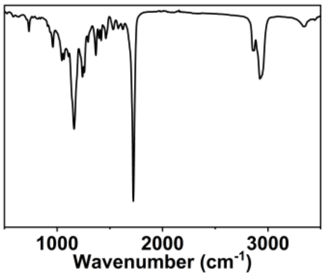

Figure S7. FTIR spectra of PU-PCL-15%. The following characteristic peaks confirm the chemical structures: 3323 cm^{-1} (ν(-NH-)), 2945 and 2865 cm^{-1} (ν(CH_3, CH_2)), 1730 cm^{-1} (ν_{as}(C=O)), 1537 cm^{-1} (δ(C-N-H)), 1463 cm^{-1} (δ(CH_2)), 1240 and 1101 cm^{-1} (ν(C-O-C)), 1043 cm^{-1} (δ(C-O-C)), 1187 cm^{-1} (ν(O-C-O)).

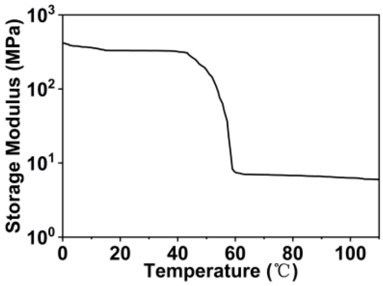

Figure S8. The DMA curve of PU-PCL-15%.

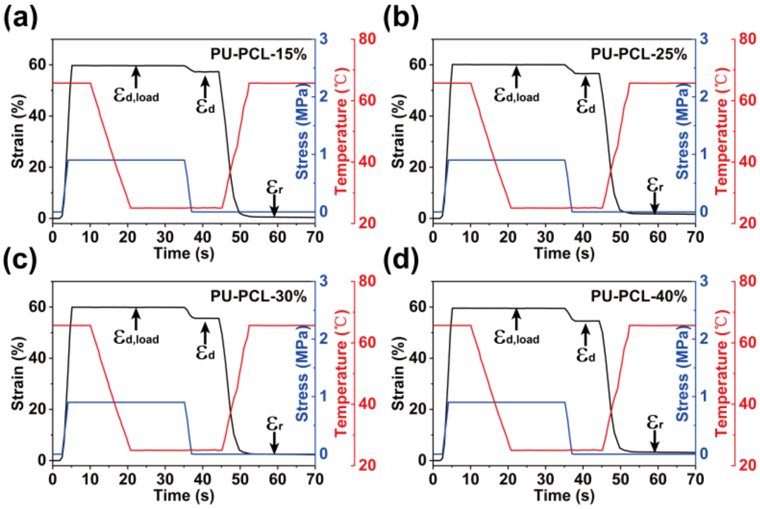

Figure S9. Thermomechanical tensile curves of (a) PU-PCL-15%, (b) PU-PCL-25%, (c) PU-PCL-30%, and (d) PU-PCL-40%.

The R_f and R_r of the PU-PCL sheets were calculated according to the following formulas:

$$R_f = \frac{\varepsilon_d}{\varepsilon_{d,load}} \times 100\%, \qquad \text{S1}$$

$$R_r = \frac{\varepsilon_d - \varepsilon_r}{\varepsilon_d} \times 100\%, \qquad \text{S2}$$

where $\varepsilon_{d,load}$, ε_d, and ε_r represent the maximum strain under load, the shape-fixed strain, and the recovered strain, respectively.

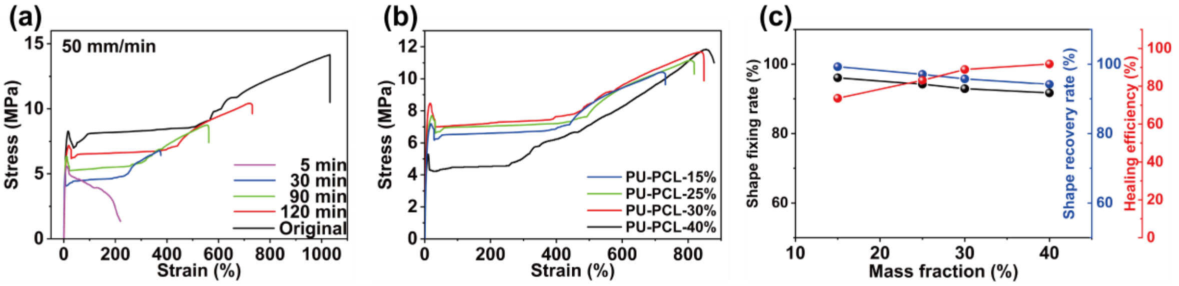

Figure S10. (a) The stress-strain curves of PU-PCL-15% during a self-healing process. (b) The stress-strain curves of PU-PCL sheets after 2 h self-healing. (c) The variations in R_f, R_r, and healing efficiency of PU-PCL sheets as a function of PCL content.

7. Preparation of graphene oxide (GO)

First, 1.0 g graphite and 0.50 g sodium nitrate were inserted into a flask at 0 °C, and 23 mL H_2SO_4 (98%) was added to the flask slowly under vigorous stirring. After 10 min, 3.01 g potassium manganese peroxide was added, and the mixed reaction system became light green and viscous within 30 min. The reaction system was stirred at 35 ^{o}C for 90 min resulting in a green suspension, and then 46 mL water was slowly added (over 10 min) to the reaction system. The reaction lasted for 15 min at 98 ^{o}C. The mixture was then cooled to room temperature, and then 140 mL water and 10 mL hydrogen peroxide (30%) were added to the reaction system. After 10 min, 40 mL HCl (35%) was added to the solution. Insoluble powders settled on the bottom of the flask. The resulting precipitate was subjected to multiple suction filtrations to remove the excess acid, then centrifuged, and washed to remove by-products. After this, the obtained neutral graphene oxide was dispersed in water and sonicated for 30 min and then centrifuged at 8000 $r{\cdot}min^{-1}$ for 30 min.

8. Preparation of PEDOT:PSS-rGO electrode

PEDOT:PSS and rGO (20 wt%) were dispersed in a mixed solution of water/ethanol/dimethyl sulfoxide (1:1:0.06) by sonication. A 10 mL composite suspension aliquot was cast onto a PU-PCL substrate and dried at 80 ^{o}C for 30 min to obtain PEDOT:PSS-rGO electrodes.

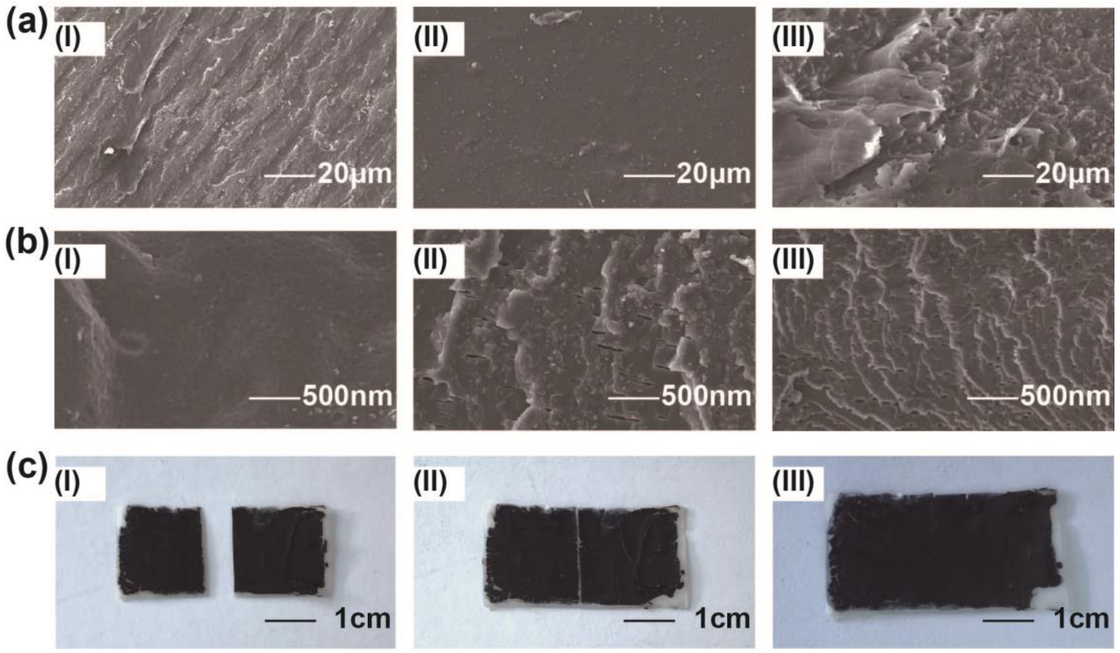

Figure S11. (a) The SEM images of (I) PU-PCL, (II) PEDOT:PSS, and (III) PEDOT:PSS-rGO. (b) The cross-sectional SEM images of (I) PU-PCL, (II) PEDOT:PSS, and (III) PEDOT:PSS-rGO. (c) The optical images of the PEDOT:PSS-rGO electrode during the self-healing process.

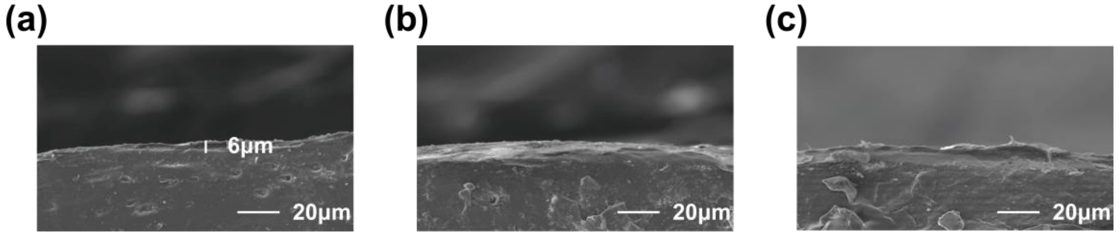

Figure S12. The cross-sectional SEM images of (a) the original PEDOT:PSS-rGO electrode, (b) PEDOT:PSS-rGO electrode after 10 “U” shape-editing cycles, and (c) PEDOT:PSS-rGO electrode after 5 cutting/self-healing cycles.

9. Preparation of Supercapacitor

An all-solid supercapacitor was fabricated by sandwiching the hydrogel-5-8 electrolyte between two PEDOT:PSS-rGO electrodes on PU-PCL-15% substrates. The supercapacitors were encapsulated with Norland Optical Adhesive-63 (NOA-63) adhesive. The supercapacitor was then irradiated with a 250 W UV lamp (λ = 365 nm) for 5 min at a distance of 12 cm to

solidify the NOA-63 and form a waterproof layer. NOA-63 contains mercapto acrylate and triallyl isocyanurate. When NOA-63 is irradiated by UV light, the photoinitiator benzoin ether is cleaved to form alkoxy radicals initiating the free radical polymerization of the mercapto acrylate. The triallyl isocyanurate is a crosslinker that can react with the thiol forming -NCOS- groups to solidify the adhesive. The NOA-63 adhesive is hydrophobic that hence can prevent water evaporation. When it was heated up at > 60 ^{o}C, the NOA-63 adhesive softens and polymer segments can migrate and entangle in this state. The residual thiol and isocyanurate groups can react to further form 3D crosslinking structures. Therefore the NOA-63 adhesive shows the self-healing capability at a high temperature.

10. Characterization of supercapacitors

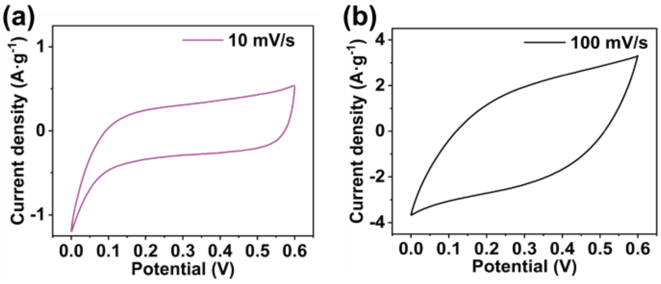

Figure S13. The CV curves of a supercapacitor at the scan rates of (a) 10 mV·s^{-1} and (b) 100 mV·s^{-1}.

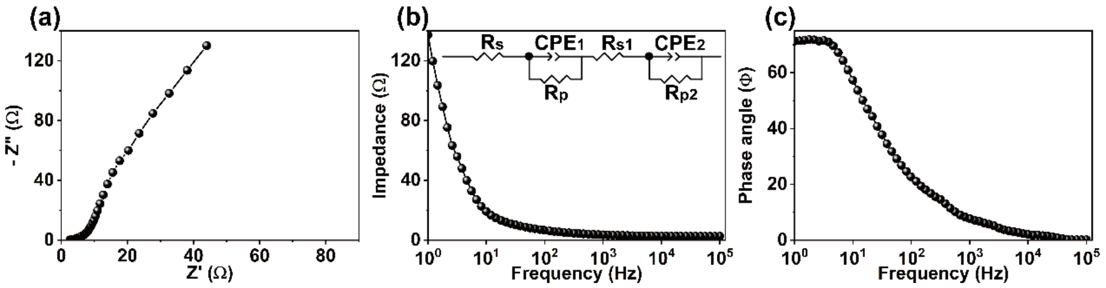

Figure S14. (a) The Nyquist plot of a supercapacitor. (b) The impedance of the supercapacitor as a function of frequency (inset shows the equivalent circuit). (c) The Bode phase angle plot of a supercapacitor.

The values of the specific capacitance (C_{sp}, F·g^{-1}), energy density (E, Wh·kg^{-1}), and power density (P, W·kg^{-1}) of the supercapacitors were calculated according to the following formulas:

$$C_{sp} = \left(\int IdU\right)/2v\Delta U, \quad \text{S3}$$

$$C_{sp} = I \triangle t / \triangle U, \quad \text{S4}$$

$$E = [C_{sp} \times V^2] \times (1000/3600), \quad \text{S5}$$

$$P = E/\Delta t, \quad \text{S6}$$

where I is the response current (A), *v* is the scan rate (mV/s), V = ΔU is the potential window, and Δt is the discharging time (s).

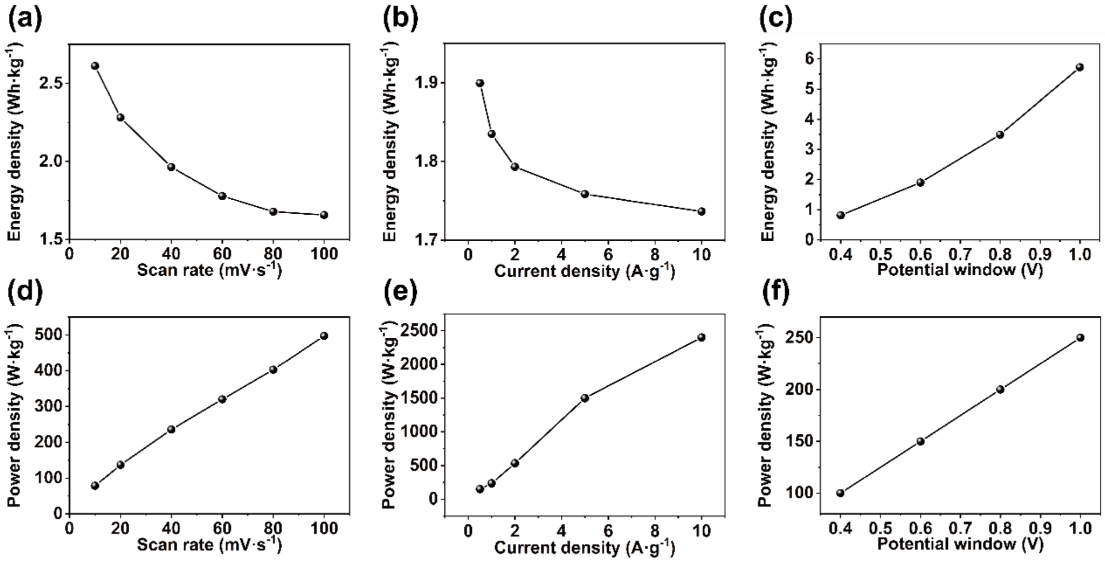

Figure S15. (a–c) The energy density as the functions of the scan rate, current density, and potential window, respectively. (d–f) The power density as the functions of scan rate, current density, and potential window.

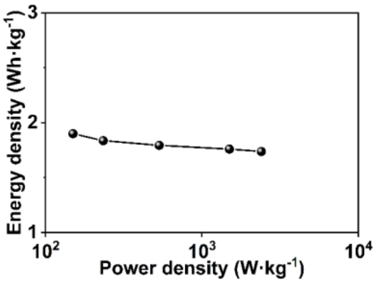

Figure S16. The Ragone plot of energy density and power density of a supercapacitor.

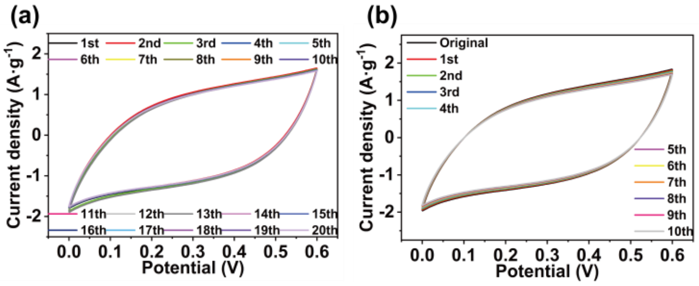

Figure S17. (a) The CV curves of supercapacitors during 20 “U” shape-editing cycles. (b) The CV curves of supercapacitors during 10 cutting/self-healing cycles.

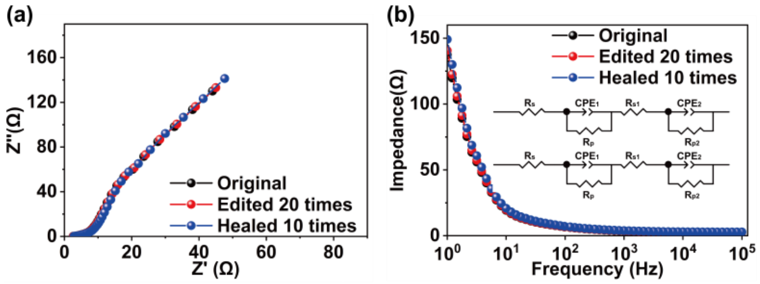

Figure S18. (a) The Nyquist plot of the original supercapacitor, the supercapacitor after 20 “U” shape-editing and 10 cutting/self-healing cycles. (b) The impedance of the original supercapacitor, the supercapacitor after 20 “U” shape-editing and 10 cutting/self-healing cycles as a function of frequency.

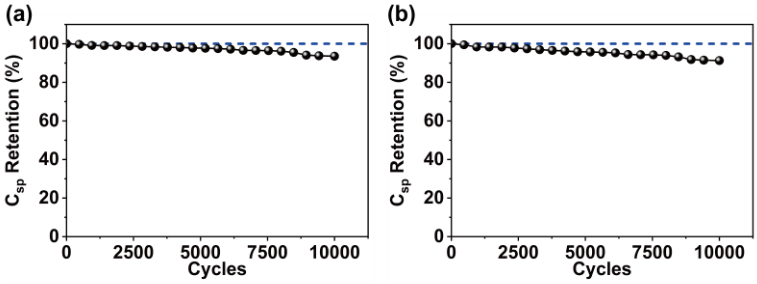

Figure S19. (a) Capacitance retention of a supercapacitor after 20 “U” shape-editing during 10,000 charging and discharging cycles. (b) Capacitance retention of a supercapacitor after 10 cutting/self-healing during 10,000 charging and discharging cycles.

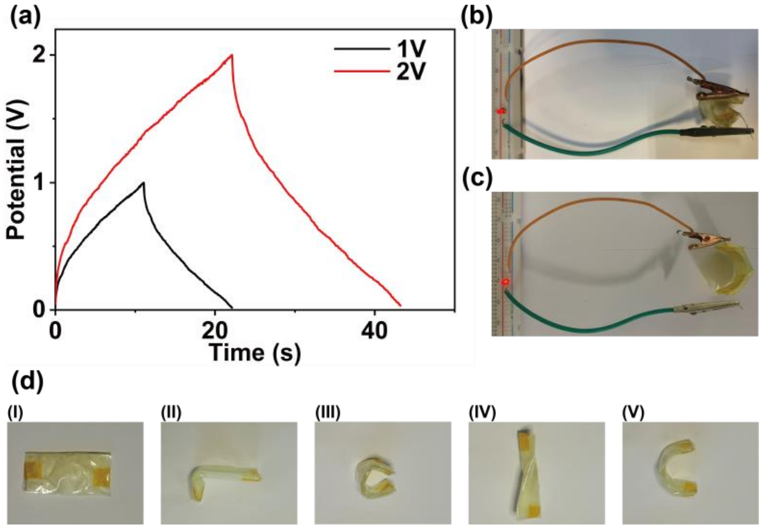

Figure S20. (a) The GCD measurements for different potential windows at a current density of 5 A·g^{-1}. Photographs of an electronic watch powered by a supercapacitor in (b) "ring" and (c) "U" shapes. (d) Photographs of supercapacitors in (I) "flat", (II) "L", (III) "ring", (IV) "spiral" and (V) "U" shapes.

References:

[1] Y. Wang, X. Liu, S. Li, T. Li, Y. Song, Z. Li, W. Zhang, J. Sun, *ACS Appl. Mater. Interfaces* **2017**, *9*, 29120.

[2] Y. Li, D. Wang, J. Wen, J. Liu, D. Zhang, J. Li, H. Chu, *Adv. Funct. Mater.* **2021**, *31*, 2011259.

[3] C. Chen, H. Qin, H. Cong, S. Yu, *Adv. Mater.* **2019**, *31*, 1900573.

[4] Q. Zhang, X. Liu, X. Ren, F. Jia, L. Duan, G. Gao, *Chem. Mater.* **2019**, *31*, 5881.

[5] S. H. Kim, S. Jung, I. S. Yoon, C. Lee, Y. Oh, J.-M. Hong, *Adv. Mater.* **2018**, *30*, 1800109.

[6] H. Li, T. Lv, H. Sun, G. Qian, N. Li, Y. Yao, T. Chen, *Nat Commun* **2019**, *10*, 536.

[7] I. Jeon, J. Cui, W. R. K. Illeperuma, J. Aizenberg, J. J. Vlassak, *Adv Mater* **2016**, *28*, 4678.

[8] H. C. Yu, S. Y. Zheng, L. Fang, Z. Ying, M. Du, J. Wang, K. Ren, Z. L. Wu, Q. Zheng, *Adv. Mater.* **2020**, *32*, 2005171.

[9] J. Ai, K. Li, J. Li, F. Yu, J. Ma, *Int. J. Biol. Macromol.* **2021**, *172*, 66.

[10] Q. Zhang, X. Liu, L. Duan, G. Gao, *J. Mater. Chem. A* **2021**, *9*, 1835.
